# Supplementary material for: Early environments and exploration in the preschool years
Source: PLoS One. 2024 Jun 10;19(6):e0305353. doi: 10.1371/journal.pone.0305353 (PMC11164363; doi:10.1371/journal.pone.0305353)
Supplement: S1 Appendix — (PDF) [file pone.0305353.s001.pdf]

S1 Appendix

Full frequency distributions for each of the variables in our dataset, split by experiment.

Demographics

	Experiment 1	Experiment 2	Experiment 3	Experiment 4	Total
Child Age	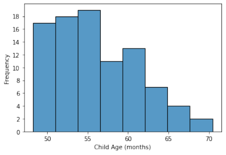<br>M = 56.25 SD = 5.16<br>Min = 48.26 Max = 70.42<br>N = 91	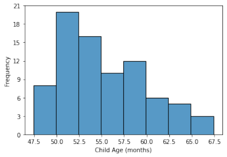<br>M = 55.26 SD = 4.83<br>Min = 47.47 Max = 67.40<br>N = 80	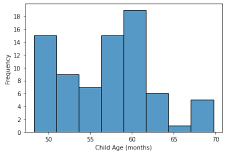<br>M = 57.06 SD = 5.30<br>Min = 48.30 Max = 69.73<br>N = 77	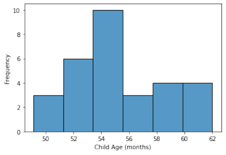<br>M = 55.33 SD = 3.46<br>Min = 49.12 Max = 62.01<br>N = 30	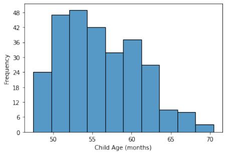<br>M = 56.09 SD = 4.98<br>Min = 47.47 Max = 70.42<br>N = 278
Child Gender	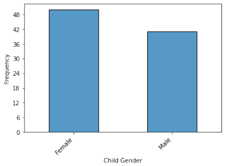<br>N = 50 Female N = 41 Male	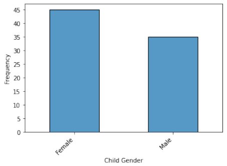<br>N = 45 Female N = 35 Male	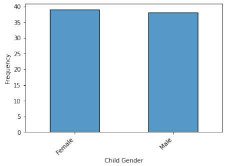<br>N = 39 Female N = 38 Male	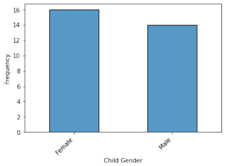<br>N = 16 Female N = 14 Male	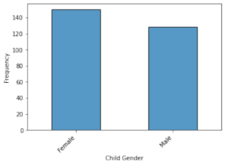<br>N = 150 Female N = 128 Male
Median Income (Home Zip Code)	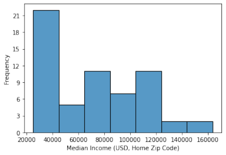<br>M = 71,973.03 SD = 37,540.41<br>Min = 25,192.00 Max = 163,403.00<br>N = 60	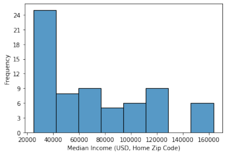<br>M = 73,209.09 SD = 41,737.74<br>Min = 25,192.00 Max = 163,403.00<br>N = 68	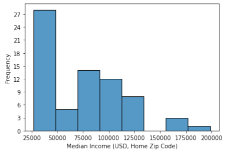<br>M = 74,494.61 SD = 38,467.40<br>Min = 26,970.00 Max = 198,625.00<br>N = 71		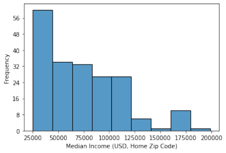<br>M = 73,295.06 SD = 39,161.26<br>Min = 25,192.00 Max = 198,625.00<br>N = 199
Modal Education (Home Zip Code)	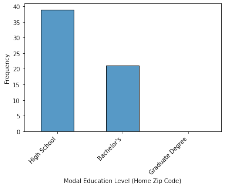<br>N = 39 High school N = 21 Bachelor's	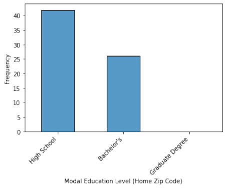<br>N = 42 High school N = 26 Bachelor's	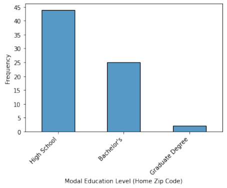<br>N = 44 High school N = 25 Bachelor's N = 2 Graduate Degree		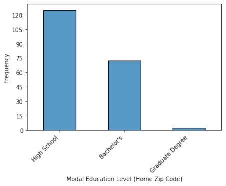<br>N = 125 High school N = 72 Bachelor's N = 2 Graduate Degree

	Experiment 1	Experiment 2	Experiment 3	Experiment 4	Total
Self-reported Parental Income	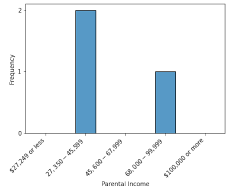<br>N = 2 $27,350-45,599<br>N = 1 $68,000-99,999	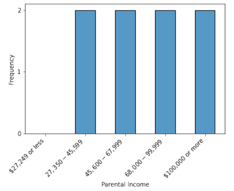<br>N = 2 $27,350-45,599<br>N = 2 $45,600-67,999<br>N = 2 $68,000-99,999<br>N = 2 $100,000 or more	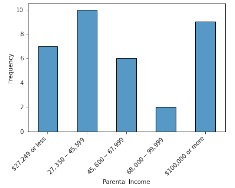<br>N = 7 $27,249 or less<br>N = 10 $27,350-45,599<br>N = 6 $45,600-67,999<br>N = 2 $68,000-99,999<br>N = 9 $100,000 or more		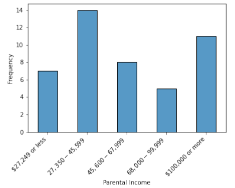<br>N = 7 $27,249 or less<br>N = 14 $27,350-45,599<br>N = 8 $45,600-67,999<br>N = 5 $68,000-99,999<br>N = 11 $100,000 or more
Self-reported Parental Education	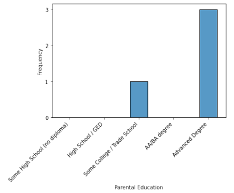<br>N = 1 Some college<br>N = 3 Advanced degree	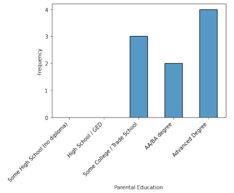<br>N = 3 Some college<br>N = 2 AA/BA degree<br>N = 4 Advanced degree	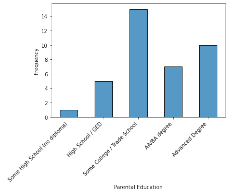<br>N = 1 Some high school<br>N = 5 High school / GED<br>N = 15 Some college<br>N = 7 AA/BA degree<br>N = 10 Advanced degree		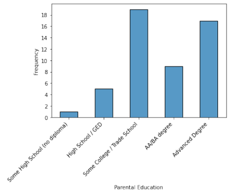<br>N = 1 Some high school<br>N = 5 High school / GED<br>N = 19 Some college<br>N = 9 AA/BA degree<br>N = 17 Advanced degree
Child Race / Ethnicity	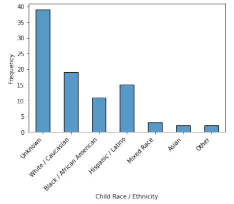<br>N = 39 Unknown<br>N = 19 White<br>N = 11 Black<br>N = 15 Hispanic<br>N = 3 Mixed<br>N = 2 Asian<br>N = 2 Other	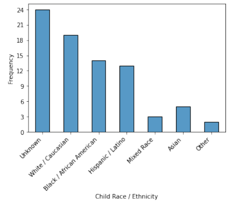<br>N = 24 Unknown<br>N = 19 White<br>N = 14 Black<br>N = 13 Hispanic<br>N = 3 Mixed<br>N = 5 Asian<br>N = 2 Other	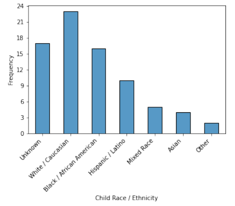<br>N = 17 Unknown<br>N = 23 White<br>N = 16 Black<br>N = 10 Hispanic<br>N = 5 Mixed<br>N = 4 Asian<br>N = 2 Other		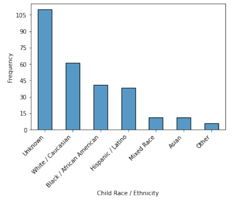<br>N = 110 Unknown<br>N = 61 White<br>N = 41 Black<br>N = 38 Hispanic<br>N = 11 Mixed<br>N = 11 Asian<br>N = 6 Other

Play (normalized)

	Experiment 1	Experiment 2	Experiment 3	Experiment 4	Total
Total Playtime	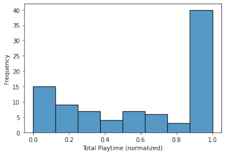<br>M = .620 SD = .376<br>Min = 0 Max = 1<br>N = 91	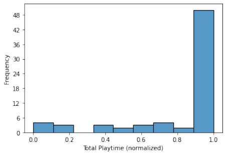<br>M = .828 SD = .306<br>Min = 0 Max = 1<br>N = 71	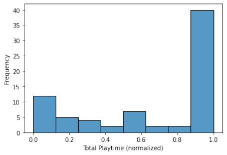<br>M = .681 SD = .385<br>Min = 0 Max = 1<br>N = 74	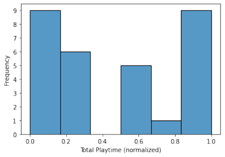<br>M = .476 SD = .400<br>Min = 0 Max = 1<br>N = 30	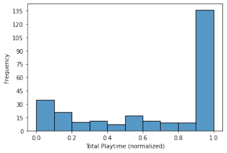<br>M = .676 SD = .378<br>Min = 0 Max = 1<br>N = 266
Key Function Playtime	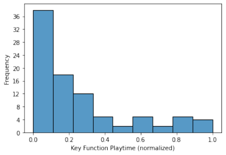<br>M = .259 SD = .274<br>Min = 0 Max = 1<br>N = 91	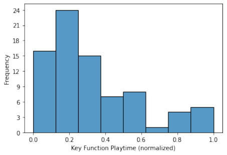<br>M = .328 SD = .262<br>Min = 0 Max = 1<br>N = 80	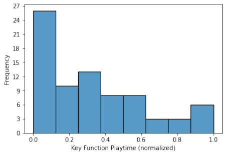<br>M = .326 SD = .295<br>Min = 0 Max = 1<br>N = 77		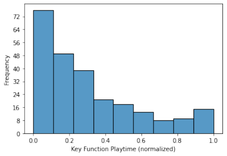<br>M = .302 SD = .278<br>Min = 0 Max = 1<br>N = 248
Unique Actions – Minute 1	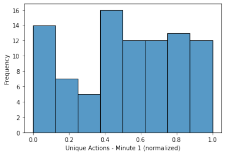<br>M = .516 SD = .316<br>Min = 0 Max = 1<br>N = 91				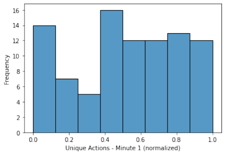<br>M = .516 SD = .316<br>Min = 0 Max = 1<br>N = 91
Unique Actions – Total	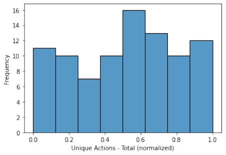<br>M = .519 SD = .293<br>Min = 0 Max = 1<br>N = 89	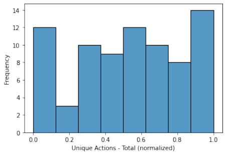<br>M = .538 SD = .313<br>Min = 0 Max = 1<br>N = 78	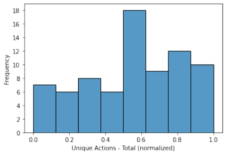<br>M = .548 SD = .284<br>Min = 0 Max = 1<br>N = 76	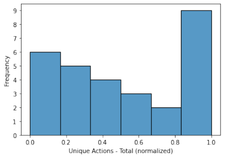<br>M = .507 SD = .333<br>Min = 0 Max = 1<br>N = 29	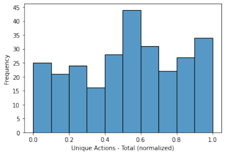<br>M = .531 SD = .299<br>Min = 0 Max = 1<br>N = 272

	Experiment 1	Experiment 2	Experiment 3	Experiment 4	Total
Proportion of Key Function Play	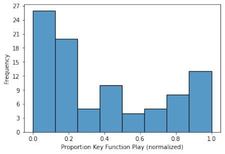<br>M = .397 SD = .338<br>Min = 0 Max = 1<br>N = 91	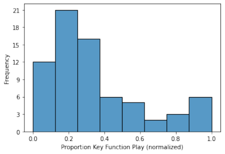<br>M = .347 SD = .267<br>Min = 0 Max = 1<br>N = 71	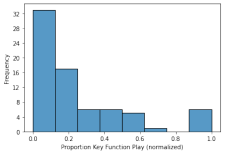<br>M = .245 SD = .281<br>Min = 0 Max = 1<br>N = 74		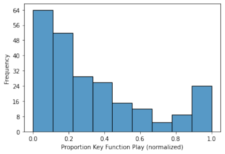<br>M = .334 SD = .306<br>Min = 0 Max = 1<br>N = 236
Variability Rate	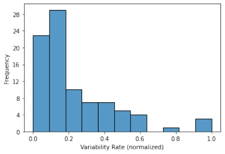<br>M = .238 SD = .221<br>Min = 0 Max = 1<br>N = 89	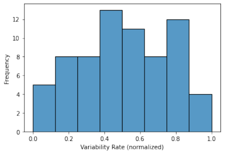<br>M = .517 SD = .269<br>Min = 0 Max = 1<br>N = 69	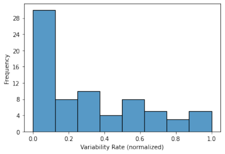<br>M = .318 SD = .301<br>Min = 0 Max = 1<br>N = 73	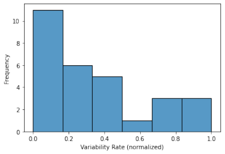<br>M = .340 SD = .316<br>Min = 0 Max = 1<br>N = 29	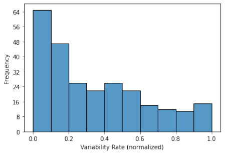<br>M = .346 SD = .289<br>Min = 0 Max = 1<br>N = 260
