# Supplementary material for: Early environments and exploration in the preschool years
Source: PLoS One. 2024 Jun 10;19(6):e0305353. doi: 10.1371/journal.pone.0305353 (PMC11164363; doi:10.1371/journal.pone.0305353)
Supplement: S2 Appendix — (PDF) [file pone.0305353.s002.pdf]

S2 Appendix

A summary of all of the analyses performed on our compiled dataset.

Primary correlations

	**Total Playtime**	**Key Function Playtime**	**Unique Actions – Minute 1**	**Unique Actions – Total**	**Proportion of Key Function Play**	**Variability Rate**	**α**
Age	$r(264) = .069$<br>$p = .261$	$r(246) = -.035$<br>$p = .582$	$r(89) = .142$<br>$p = .179$	$r(270) = .168$*<br>$p = .005$	$r(234) = -.089$<br>$p = .175$	$r(258) = .052$<br>$p = .407$	.008
Gender	$t(264) = -.450$<br>$p = .653$	$t(246) = .771$<br>$p = .441$	$t(89) = 1.76$<br>$p = .081$	$t(270) = .493$<br>$p = .623$	$t(233) = -.748$†<br>$p = .455$	$t(258) = .926$<br>$p = .355$	.008
Income	$r(187) = -.097$<br>$p = .186$	$r(197) = -.044$<br>$p = .537$	$r(58) = -.136$<br>$p = .300$	$r(192) = -.193$*<br>$p = .007$	$r(187) = .113$<br>$p = .123$	$r(183) = -.032$<br>$p = .663$	.008
Education	$t(187) = 1.07$<br>$p = .286$	$t(197) = .348$<br>$p = .728$	$t(58) = .689$<br>$p = .493$	$t(192) = 1.60$<br>$p = .112$	$t(119) = -1.42$†<br>$p = .158$	$t(183) = -.235$<br>$p = .815$	.008

Follow-up partial correlations (controlling for age in months)

	**Total Playtime**	**Key Function Playtime**	**Unique Actions – Minute 1**	**Unique Actions – Total**	**Proportion of Key Function Play**	**Variability Rate**	**α**
Income	$r(187) = -.102$<br>$p = .166$	$r(197) = .042$<br>$p = .556$	$r(58) = -.156$<br>$p = .237$	$r(192) = -.206$*<br>$p = .004$	$r(187) = .118$<br>$p = .107$	$r(183) = -.034$<br>$p = .650$	.008

*Significant at the designated α level.
~Marginally significant at the designated α level.
†Assumption of equal sample variance violated; heteroscedasticity assumed.

Correlations split by SES

		Unique Actions – Total	α
**Proportion of Key Function Play**	**Low Modal Education (Home Zip Code)**	*r*(119) = -.339* $p < .001$	.025
	**High Modal Education (Home Zip Code)**	*r*(62) = -.070 $p = .583$	.025

Follow-up regression

	Unique Actions – Total	α
**Model**	$F(3,181) = 4.93$*; adj. $R^2 = .060$; $p = .003$	.05

Predictor	*B*	*t*	*p*	α
Intercept	0.77	9.07*	<.001	.05
Proportion of Key Function Play	-.555	-2.74*	.007	.05
Education	-.100	-1.69~	.093	.05
Interaction	.231	1.72~	.088	.05

Correlation matrix between all play measures

	**Total Playtime**	**Key Function Playtime**	**Unique Actions – Minute 1**	**Unique Actions – Total**	**Proportion of Key Function Play**	**Variability Rate**
**Total Playtime**		$r(246) = .396$* $p < .001$	$r(89) = .112$ $p = .292$	$r(276) = .497$* $p < .001$	$r(246) = -.157$* $p = .015$	$r(276) = -.359$* $p < .001$
**Key Function Playtime**			$r(89) = -.568$* $p < .001$	$r(246) = .157$* $p = .015$	$r(246) = .583$* $p < .001$	$r(246) = -.139$* $p = .034$
**Unique Actions – Minute 1**				$r(89) = .722$* $p < .001$	$r(89) = -.853$* $p < .001$	$r(89) = .295$* $p = .005$
**Unique Actions – Total**					$r(246) = -.327$* $p < .001$	$r(276) = .201$* $p = .001$
**Proportion of Key Function Play**						$r(246) = .049$ $p = .457$
**Variability Rate**						

Play measures were generally correlated with each other. Note that (a) Unique Actions – Minute 1 is a subset of Unique Actions – Total, (b) Variability Rate is a function of Unique Actions – Total and Total Playtime, and (c) Proportion of Key Function Play is a function of Key Function Playtime and Total Playtime; so, correlations between these variables are not independent and should be interpreted with caution.
