# Supplementary material for: Early environments and exploration in the preschool years
Source: PLoS One. 2024 Jun 10;19(6):e0305353. doi: 10.1371/journal.pone.0305353 (PMC11164363; doi:10.1371/journal.pone.0305353)
Supplement: Attachment — Submitted filename: PONE-D-23-26158_R1_ResponseLetter.pdf [file pone.0305353.s003.pdf]

April 16th, 2024

Dear Dr. Clark,

Thank you so much for your kind and thoughtful feedback on our manuscript, “Early Environments and Exploration in the Preschool Years” (PONE-D-23-26158), and for taking the time to also serve as a reviewer given the difficulty of securing additional reviews. We understand it has been extremely challenging to recruit reviewers globally, and we sincerely appreciate the effort you have put into this process.

We are very grateful for the opportunity to revise our manuscript. We have read carefully through your comments and that of the Reviewer and have made changes throughout the manuscript to address the feedback. We believe these changes have significantly improved the paper.

We are also including an updated funding statement in this cover letter, as per your request:

> This work was funded by an Early Career Research Fellowship from the Jacobs Foundation to EB (https://jacobsfoundation.org/), and a Scholar Award in Understanding Human Cognition from the James S. McDonnell Foundation to EB (https://www.jsmf.org/). IB is supported by a Mind Brain Behavior Postdoctoral Fellowship from Harvard University (https://mbb.harvard.edu/) and a STEM Education Postdoctoral Research Fellowship from the National Science Foundation (Award Number: 2327447). There was no additional external funding received for this study. The funders had no role in study design, data collection and analysis, decision to publish, or preparation of the manuscript.

Below, please find our point-by-point response to your and the Reviewer’s comments. Changes directly made to the manuscript as a result are highlighted in blue text.

We look forward to hearing from you, and thank you again for this opportunity.

Sincerely,

Drs. Ilona Bass & Elizabeth Bonawitz

Response to Editor’s Comments

===========

> **Comment:** Nicely done article, thank you for allow us to review it. I have only one review for you as I was unable to obtain a second review. This new analysis of your past data collection shows

some potentially important information that can be. used in many areas, including. Education, Recreation, and Parental Interventions.

Response: Thank you so much for the kind words. We are delighted that you find this contribution important.

===========

Comment: I note one thing that I will ask you to change. You have a don't at the top of your Discussion section.

Response: Thank you for catching this contraction. We have corrected this and two others that appeared in the manuscript (Pg. 8: “I’m”; Pg. 19: “what’s”).

Response to Reviewer #1’s Comments

===========

Comment: This manuscripts presents a relatively novel analysis that helps researchers address differences in methodologies and still explore underlying patterns in the larger data sets. The focus of the study, development of exploratory play behavior is valuable both for understanding cognitive development and for educational and curricular applications.

Response: Thank you for these kind words; we are glad you found the manuscript valuable!

===========

Comment: 1. The methods section notes that in some experiments children received pedagogical demonstration and in others a pedagogical direction - did this occur in all 4 experiments, and if not how was the toy introduced?

Response: We apologize for the lack of clarity. There was one experiment that did not involve a pedagogical cue (“Experiment 4” in S1 Appendix, N = 30 children). The other three experiments all used pedagogical demonstrations. In addition to clarifying this point, we now also describe how the toy was presented to children in absence of a pedagogical cue:

Pg. 8: If a pedagogical cue is not used, the experimenter presents the toy to the child in a more neutral manner (e.g., “Look at this toy! You can play with it to figure out how it works!”). For the data included in this analysis, three of the experiments (N = 248) included a pedagogical demonstration, while one (N = 30) did not involve any kind of pedagogical cue.

===========

> **Comment:** 2. More information on the context of the experiments would be useful - how many people in the room, were there other toys or objects in the room, etc.

Response: Thank you for this note. In general, children in these tasks are tested in quiet spaces where there is minimal distraction from people or objects not involved in the study. We have added these details to the manuscript:

Pg. 7: For all of the included experiments, children were recruited from and tested at various local preschools and daycare centers. The studies were run in relatively quiet locations within the testing sites (e.g., an empty teachers' lounge), to ensure that distractions from people or objects not involved in the study were kept to a minimum.

============

> **Comment:** 3. The age range was just shy of age 4 to just shy of age 6, were some of the 5-year-olds in Kindergarten, Transitional Kindergarten, or PreK? While you note that age differences could also reflect exposure to formal school, this point is under-emphasized. If not, a central question is whether more formal school experience informs exploratory play behavior that differ from more informal daycare or preschool settings. (what were the preschool/day care settings like - were they play based, academic, etc.)

Response: Thank you for raising these important questions. All of the children in these experiments were recruited from and tested at preschools and daycare centers, including the older children. We now note in the General Discussion how, within these preschool contexts, age might itself serve as a proxy for other variables of interest:

Pg. 15: It is also possible that age itself serves as a proxy for other potentially relevant constructs. For instance, children in these studies were all recruited from preschools and daycares, but they were as young as 3 years 11 months, and as old as 5 years 10 months. It could be the case that these older children had been enrolled in preschool for much longer than the younger children; thus, it would be difficult to disentangle the role of age from experience with these informal educational settings. Understanding the precise role of development in children's exploration is of course an open question for future work, but an exciting one nonetheless.

We do not have data on the degree to which the many preschools and daycares used for recruitment were play-based or academic. But, we believe this would be an exciting question for future work to pursue. We now discuss this important point in the General Discussion:

Pg. 17-18: For example, we were not able to capture any qualities of the preschool or daycare environments that these children were attending when they participated in their respective studies.

The degree to which such learning environments are more academically-focused versus play-based, for instance, could have influenced how children approached these novel toy tasks. Moreover, interactions with preschool teachers are some of the first formal pedagogical experiences children have. Qualities of these interactions could also reasonably affect how children interpret lightly scaffolded play moments from a new "teacher" (i.e., an experimenter), especially in the testing context of their preschool [46]... Understanding how exactly these early experiences coalesce to inform how children approach new opportunities for learning is a deep and fundamental question in child development, to be sure, and would make for exciting future work.

============

Comment: 4. I recognize that you wanted to control for variations in methodology across the different experiments, but did children's exploratory play behavior differ by prompt, controlling for age and income?

Response: Yes, the original studies for which these data were normed and combined included many different kinds of experimental manipulations, for which prompts did have an effect, even controlling for age. (Some studies did not explore income, as this factor was not recorded directly for those studies.) These separate papers have been previously published and are not the primary question of interest here. It is precisely because these prompts had differing effects that we transformed the data, norming exploration measures *within condition* to allow us to marginalize out any potential role of the prompts explored in the original experimental designs. We see this may have been confusing in the manuscript and have added the additional section header to highlight this important control:

Pg. 9-10: **Marginalizing out role of condition**

One concern with the data is that different experimental manipulations would affect these different play measures (in fact, this was predicted by the original study designs). To control for this, after performing outlier analyses, we min-max normalized all values (i.e., re-scaled quantities to be on a scale from 0 to 1, where 0 represents the minimum, and 1 represents the maximum) for each variable, within each experimental condition. Marginalizing out all effects of condition and experiment prior to conducting any analyses ensured that any effects we saw in our data could not be attributed to differences in the studies themselves. All analyses reported here are conducted on the normalized data, as opposed to raw scores. See S1 Appendix for visualizations of each of these variables' normalized distributions, both overall and split by experiment.

============

Comment: 5. What are the correlations among the variations of interest?

Response: Thank you for this question. We assume you are asking for the correlations among the play variables of interest. We agree this is likely of interest to other readers and have now included this table in S2 Appendix, which we include here as well. Unsurprisingly, play measures such as Total

Playtime and Unique Actions are strongly correlated with each other, as are measures that are subsets / functions of one another.

S2 Appendix Page 3:

Correlation matrix between all play measures

	**Total Playtime**	**Key Function Playtime**	**Unique Actions – Minute 1**	**Unique Actions – Total**	**Proportion of Key Function Play**	**Variability Rate**
**Total Playtime**		$r(246) = .396^*$ $p < .001$	$r(89) = .112$ $p = .292$	$r(276) = .497^*$ $p < .001$	$r(246) = -.157^*$ $p = .015$	$r(276) = -.359^*$ $p < .001$
**Key Function Playtime**			$r(89) = -.568^*$ $p < .001$	$r(246) = .157^*$ $p = .015$	$r(246) = .583^*$ $p < .001$	$r(246) = -.139^*$ $p = .034$
**Unique Actions – Minute 1**				$r(89) = .722^*$ $p < .001$	$r(89) = -.853^*$ $p < .001$	$r(89) = .295^*$ $p = .005$
**Unique Actions – Total**					$r(246) = -.327^*$ $p < .001$	$r(276) = .201^*$ $p = .001$
**Proportion of Key Function Play**						$r(246) = .049$ $p = .457$
**Variability Rate**						

Play measures were generally correlated with each other. Note that (a) Unique Actions – Minute 1 is a subset of Unique Actions – Total, (b) Variability Rate is a function of Unique Actions – Total and Total Playtime, and (c) Proportion of Key Function Play is a function of Key Function Playtime and Total Playtime; so, correlations between these variables are not independent and should be interpreted with caution.

============

Comment: 6. The discussion section needs to acknowledge that you only had 2 of 24 correlations be statistically significant;

Response: Thank you for raising the important statistical limitations of this work, which we now address in the General Discussion:

Pg. 18: Second, we note the statistical limitations of this work… It is important to note that while we did correct for familywise error in our analyses, few of these analyses actually yielded significant results, calling into question the true sizes of these effects. Nevertheless, we are hopeful that the relationships we describe here are sufficiently meaningful to have raised new theoretical opportunities for investigation.

============

Comment: 7. The discussion section needs to more strongly emphasize that results in lab-like settings do not necessarily reflect messy everyday life, where children likely are not often handed a single toy to explore in a relatively (I assume) empty and quiet room.

Response: We completely agree with the Reviewer that lab-based studies are necessarily limited in their ecological validity, particularly when it comes to such organic activities as play and exploration. We now raise this point in the General Discussion:

Pg. 18-19: Finally, we acknowledge limitations in the generalizability of our results… We opted for high experimental control in these studies, which often trades off with the ecological validity of the results. To what extent does children's play behavior in these studies actually map onto playful learning and exploration in the messy environments of real life? Indeed, researchers have long struggled to even define "play" [48], which makes it a notoriously difficult behavior to study in development. Designing studies that opt for more ecological validity is one way in which future work could further connect the current findings to children's play in the real world.

============

Comment: 8. Some discussion of how and when exploratory play

Response: We regret that this final comment from the Reviewer seems to have been cut off. We suspect it was related to the comment above, requesting additional details regarding how and when exploratory play might differ in settings with greater ecological validity. We hope the additional discussion noted above addresses the Reviewer's intended comment. However, if the Reviewer wishes to restate this comment in their review of the revised manuscript, we would be more than happy to address it then.

We once again want to thank the Editor and the Reviewer, both for their positive assessments and for their many good points. We hope our revisions have addressed the concerns, and believe the manuscript is now much stronger as a result.
